# Need for therapeutic interventions as a predictor of mortality in intensive care

**DOI:** 10.1186/cc14640

**Published:** 2015-03-16

**Authors:** I Efendijev, R Raj, S Hoppu, MB Skrifvars, M Reinikainen

**Affiliations:** 1 HUS, Helsinki, Finland; 2 TAYS, Tampere, Finland; 3 PKKS, Joensuu, Finland

## Introduction

Various therapeutic interventions needed in critical care may reflect a high risk of death. We evaluated associations between commonly used interventions and hospital mortality in Finnish ICU patients.

## Methods

We retrieved data on adult patients treated in Finnish ICUs between 2003 and 2013 from the Finnish Intensive Care Consortium database. We used the Therapeutic Intervention Scoring System (TISS-76) for categorizing ICU interventions and the Simplified Acute Physiology Score (SAPS II) for quantifying severity of illness. We excluded readmissions, patients with missing outcome, SAPS II and TISS data. We also excluded very common interventions (arterial line, bolus intravenous medication), very rare ones (prevalence <1%), and interventions only applicable in specific populations (intracranial pressure monitoring, intra-aortic balloon assist). We grouped several TISS categories when applicable. We performed a backward stepwise binary logistic regression analysis with TISS items to assess the impact of each intervention on hospital mortality (expressed as odds ratio (OR) with 95% confidence intervals (CIs)). Age, admission type, and SAPS score (minus age and admission type scores) were adjusted for in the multivariate analysis.

## Results

We identified 161,134 patients eligible for analysis. The multivariate analysis showed that the highest risk for hospital mortality in all patients was associated with cardiac arrest and/or countershock, OR 2.58 (95% CI = 2.43 to 2.73), SAPS II emergency admission, OR 2.52 (95% CI = 2.32 to 2.74), vasoactive drug infusion (>1 drug), OR 1.66 (95% CI = 1.59 to 1.73) and blood transfusion (a combined TISS item), OR 1.53 (95% CI = 1.44 to 1.63). TISS items associated with the lowest risk of mortality in general population were: active anticoagulation, OR 0.51 (95% CI = 0.49 to 0.53), induced hypothermia, OR 0.68 (95% CI = 0.62 to 0.74) and measurement of cardiac output by any method, OR 0.87 (95% CI = 0.83 to 0.91). All aforementioned associations were statistically significant (*P *< 0.001). There was no notable association with mortality for pulmonary artery catheter, platelet transfusion and vasoactive drug infusion (one drug) (*P >*0.05).

## Conclusion

In this large retrospective multicenter study, the TISS item associated with the highest risk of death was cardiac arrest and/or countershock. Unexpectedly, the independent effect of emergency admission was of comparable magnitude in terms of impact on hospital mortality. Of these, in-ICU cardiac arrest might be amenable to preventive measures and should be studied further.

